# Cross-Modal Conflict Increases With Time-on-Task in a Temporal Discrimination Task

**DOI:** 10.3389/fpsyg.2019.02429

**Published:** 2019-10-31

**Authors:** András Matuz, Dimitri Van der Linden, Kristóf Topa, Árpád Csathó

**Affiliations:** ^1^Medical School, Department of Behavioural Sciences, University of Pécs, Pécs, Hungary; ^2^Department of Psychology, Education, and Child Studies, Erasmus University Rotterdam, Rotterdam, Netherlands; ^3^Institute of Psychology, University of Pécs, Pécs, Hungary

**Keywords:** time-on-task, mental fatigue, cross-modal attention, time discrimination, heart rate variability

## Abstract

The modality appropriateness hypothesis argues that the auditory modality is preferred over the visual modality in tasks demanding temporal operations; hence, we predicted that responses to visual stimuli would be more sensitive to the detrimental effect of Time-on-Task. We used a bimodal temporal discrimination task. The factors were durational congruency between the modalities and the direction of modality-transmission. Participants needed to decide the duration of the cued stimulus (visual or auditory). The first five blocks of the task lasted about 1.5 h without rest [Time-on-Task (ToT) period]. The participants then had a 12-min break followed by an additional block of trials. Subjective fatigue, reaction time, error rates, and electrocardiographic data were recorded. In the visual modality, we found an enhanced congruency effect as a function of ToT. The cost of attentional shifting was higher in the auditory modality, but remained constant, suggesting that processing of auditory stimuli is robust against the effects of fatigue. Performance did not improve after the break, indicating that the effects of fatigue could not be overcome by taking a brief break. The heart rate variability (HRV) data showed that vagal inhibition increased with ToT, but this increase was not associated with the changes in performance.

## Introduction

Cognitive or mental fatigue (hereafter fatigue) is a very common everyday phenomenon resulting, for example, from prolonged performance on cognitively demanding task (e.g., during the work day). The psychological manifestation of fatigue has multiple facets including compromised cognitive functioning and task disengagement. The latter includes a reduction in willingness to exert further cognitive effort, which is considered as one of the motivational hallmarks of fatigue (e.g., Van der Linden, 2011). Changes in cognitive performance due to fatigue have predominantly been linked to reduced efficiency of the prefrontal cortex (Lorist et al., 2005; Boksem and Tops, 2008) and prefrontally mediated cognitive functions seem to be particularly sensitive to the detrimental effects of fatigue. Fatigue has also been associated with changes in dopaminergic pathways (Stahl, 2002; Lorist et al., 2005). More recently, however, it has been suggested that a suboptimal norepinephrine level (i.e., reduced activity of the locus coeruleus norepinephrine system; LC-NE system) may be associated with increased fatigue and decreased task engagement (Hopstaken et al., 2015a,b).

Although there have been several studies addressing the biopsychological nature of fatigue, most of them examined the effects of fatigue on cognitive tasks in which performance relied largely on the visual modality. Relatively few studies have examined the fatigue sensitivity of cognitive operations associated with audition. Those that have, generally suggest that prolonged performance of auditory tasks can elicit fatigue and that the psychological consequences (e.g., task disengagement) and underlying neural mechanisms are similar to those occurring in visual paradigms (e.g., Bess and Hornsby, 2014; Alhanbali et al., 2017; Key et al., 2017; Moore et al., 2017). For example, in Moore et al.’s study (2017), mental fatigue was induced by a continuous auditory choice paradigm. Their findings were clearly in line with the existing fatigue literature, showing that the prolonged performance of an auditory task increased subjective fatigue, decreased motivation (i.e., prompted disengagement from the task), and compromised cognitive performance. In addition, like studies using visual stimuli (e.g., Boksem et al., 2005), they observed changes in electrophysiological parameters (i.e., decreased N1 EEG amplitude) indicative of reduced arousal and motivation. In summary, the findings of previous studies support the conclusion that fatigue is a general, multimodal phenomenon (Moore et al., 2017) and that continuous tasks requiring processing of visual or auditory stimuli elicit similar fatigue effects.

Nevertheless, the dominance of one particular modality over another is a well-established observation in tasks entailing conflicts or interactions between stimuli in different sensory modalities. The modality appropriateness hypothesis (Welch and Warren, 1980) postulates that, in a multimodal task context, perceptual preference is given to the sensory modality that provides the more reliable task-relevant information for an efficient performance of the actual task. In spatial tasks, therefore, individuals’ perception is dominated by visual information, whereas audition, with its greater temporal resolution, might dominate tasks where temporal judgments are more important. From a fatigue research perspective, the task-dependency of perceptual preference raises questions about how comparable the sensitivity of the different modalities to fatigue is, when there are different stimulus modalities in the same task context. One possibility is that both modalities would be affected by fatigue to the same extent, but an alternative possibility is that the higher dominance of the dominant modality increases as fatigue accumulates (see explanation below). To our knowledge, these possibilities have not been directly tested in previous studies. In general, there is a lack of studies addressing the sensitivity of cross-modal dominance under fatigue. The aim of this study, therefore, was to contribute to the fatigue literature by testing how cross-modal interactions are affected by the mental fatigue induced by prolonged performance of an attention-demanding task. Our hypothesis was derived from earlier studies suggesting that prolonged performance reduces individuals’ attentional focus and motivation to perform the task (e.g., Tanaka et al., 2014; Möckel et al., 2015; Hopstaken et al., 2016). Reductions in ability or motivation to focus attention on the task due to fatigue may result in secondary task processes being accorded lower priority. More specifically, in order to maintain performance when fatigued, people may allocate their remaining resources to the primary aspects of a task at the expense of secondary aspects (Hockey, 1997). In dual-modality tasks involving visual and auditory stimuli presented simultaneously, this would mean that, as fatigue increased, the secondary modality (i.e., the less preferred modality) received less attention, and therefore were more disturbed by the primary modality.

In order to test this hypothesis, we adapted the cross-modal temporal discrimination task used by Lukas et al. (2014) for use in a Time-on-Task paradigm. Lukas et al. (2014) examined inter-sensory bias toward audition rather than vision in a temporal judgment task. On the basis of the modality appropriateness hypothesis, they predicted that preference would be given to auditory stimuli when processing information about time. They presented auditory and visual stimuli of various durations simultaneously. On each trial, participants were required to judge the duration of the visual or the auditory stimulus; a cue preceding the trial indicated the relevant modality. The cross-modality interference effect (i.e., durational incongruence between the two modalities) and the effect of switching attention between the stimulus modalities were analyzed. In line with the modality appropriateness hypothesis, Lukas et al. (2014) found that the cross-modality interference effect (or cross-modality conflict) was larger for trials with visual targets and auditory distractors than for trials with auditory targets and visual distractors, suggesting auditory dominance in temporal judgment tasks (see also, Burr et al., 2009). In contrast, visual modality showed a benefit over audition in modality switch cost: the visual-to-auditory transition was more difficult relative to visual repetition trials than the auditory-to-visual transition relative to the auditory repetition trials.

Many previous studies of fatigue have confirmed that continuous tasks requiring high level of attentional control, such as switching tasks (e.g., Lorist et al., 2000, 2009) or selective attention tasks (e.g., Boksem et al., 2006; Csathó et al., 2012), reliably induce mental fatigue, and that such fatigue frequently (but not always) compromises individuals’ ability to perform the task. The task used by Lukas et al. (2014) requires both attentional switching and attention selection abilities; hence, it is rather demanding and we therefore expected that prolonged, continuous performance of this task (i.e., over a 1.5 h period) would elicit mental fatigue. Regarding the modality-related changes induced by fatigue, first, we predicted that vision, which is accorded lower priority than audition in this task, would generally be more sensitive to the detrimental effect of Time-on-Task. Second, we also predicted that while the interfering effect of incongruent visual stimuli on processing of the cued auditory stimuli would remain unchanged over time, the interfering effect of incongruent auditory stimuli on processing of the cued visual stimuli would increase as a function of Time-on-Task.

An important methodological element of our experiment was that after performing the task continuously for a long period, participants had a short (12 min) break before performing a final block of trials. This element was added to the design to enable us to investigate whether any fatigue-related changes that developed during the Time-on-Task period would be reversed during a short break.

Finally, the experiment was combined with measurement of heart rate variability (HRV). HRV is a psychophysiological parameter well-suited in providing evidence of an association between fatigue-sensitive psychological operations and processes of the autonomic nervous system. Vagal control over cardiac activity, measured as HRV, has been identified as an important marker of fatigue, and HRV can predict the concurrent drop in cognitive performance (e.g., Fairclough and Houston, 2004; Segerstrom and Nes, 2007; Tran et al., 2009; Mizuno et al., 2011, 2014; Gergelyfi et al., 2015). In addition, because there is a relationship between LC-NE activation and vagal activity (Mather et al., 2017), the idea that the LC-NE systems plays a role in mental fatigue (Hopstaken et al., 2015a,b) was considered reason to analyze HRV.

## Materials and Methods

### Participants

Twenty-five undergraduate and postgraduate students participated. The data of two participants were excluded from analysis due to a computer error during the experiment. Hence, the final dataset consisted of data from 23 participants (15 women and 8 men; aged between 19 and 25 years; *M* = 21.43; SD = 1.73). The sample size was calculated based on earlier studies and a series of power analyses conducted by Gpower 3.1 (Faul et al., 2007). Specifically, the minimum sample size to ensure the statistical power of the main effects of durational congruency and modality, as well as the interaction of these factors were estimated based on Lukas et al. (2014), experiment 1. These effects and interactions were highly robust with effect sizes (i.e., $\eta_{p}^{2}$) ranged between 0.27 and 0.98. By applying these values, the recommended minimum sample size for these effects and interactions was less than 10 to achieve a power level of 90% and alpha <0.05. In addition, to attain the sufficient power of the effect of Time-on-Task (ToT) on performance measures and HRV, we considered recent ToT studies addressing mental workload (e.g., Hopstaken et al., 2015a; Hidalgo-Muñoz et al., 2018; Reteig et al., 2019) as well as our earlier studies conducted with protocols similar to the current study (Csathó et al., 2012, 2013, 2015). Calculating with the effect sizes reported in these papers, about 19 participants were recommended as a minimum sample size to detect ToT-related effects (power = 90% and alpha <0.05). In sum, the final sample of 23 participants had the appropriate statistical power to detect the effects we aimed to examine.

According to their self-reports, 21 participants were right-handed, and none had a history of neurological disease or mental disorder. All reported normal or corrected-to-normal vision, normal hearing and none were taking medication. All participants provided written consent. To avoid a large mismatch between participants’ general daytime activity and the time of the test the self-assessment version of the Morningness-Eveningness Questionnaire, (Self-Assessment version; MEQ-SA) was used to assess preferred time for activity and participants with a preference for being active in the afternoon and evening hours (i.e., an evening type chronotype) were always tested in the afternoon. Five of the participants were “evening-types” (MEQ-SA score ≤ 41) and 18 were “intermediate-types” (MEQ-SA score 42 58) (Terman et al., 2001).

### Task and Stimuli

Participants performed a modified version of the cross-modal switching task developed by Lukas et al. (2014). The script used to control stimulus presentation and record responses was written in PsychoPy (version 1.85.6 for Windows: Peirce, 2007, 2009).

**Figure 1:**
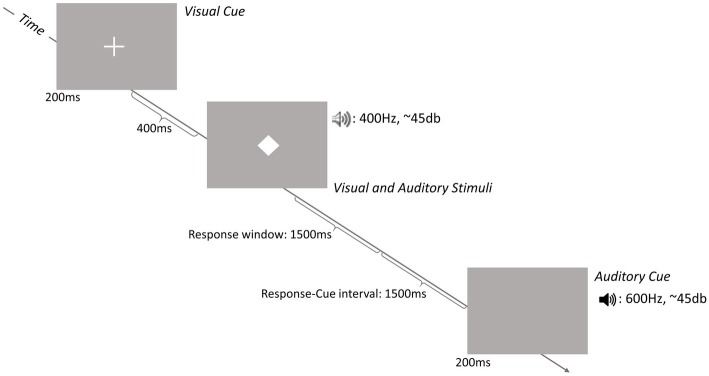
Schematic representation of the sequence of a trial. Each trial started with the onset of either an auditory or a visual cue followed by the simultaneous presentation of an auditory stimulus and a visual stimulus. Participants were asked to decide if the stimulus of the cued modality (i.e., the stimulus in the modality corresponding to the modality of the cue) was presented for a short (100 ms) or a long (300 ms) time period.

Figure 1 schematizes the sequence of a trial. At the beginning of each trial, a visual or an auditory cue was presented for 200 ms to indicate the trial-relevant stimulus modality. The visual cue was a white cross (1.5 cm × 1.5 cm) with a visual angle of 1.25° presented on a gray background at the center of the screen. The auditory cue was a 600 Hz tone presented *via* standard loudspeakers at a volume of approximately 45 dB. The number of consecutive trials with the same cue modality (i.e., repetition trials) varied between 2 and 5. In each trial, the cue was followed by simultaneous presentation of visual and auditory stimuli; stimulus presentation lasted either 100 (short) or 300 ms (long).

**Figure 2:**
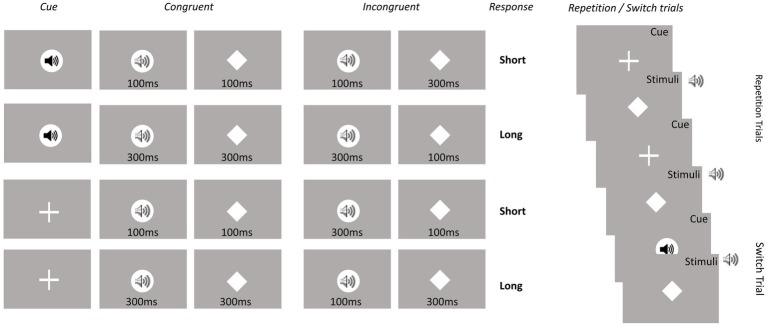
Experimental conditions. Overall, eight conditions were included based on the eight possible cue-stimulus-modality transition combinations. Conditions represent Modality (visual vs. auditory), Modality transition (switch vs. repetition) and Congruency (congruent vs. incongruent).

On congruent trials, the duration of the auditory and visual stimuli was the same and on incongruent trials, the durations of the two stimuli were different (see Figure 2). The visual stimulus was a centrally presented white diamond (1.5 cm × 1.5 cm) with a visual angle of 1.25°. The auditory stimulus was a 400 Hz tone of the same volume as the auditory cue. Participants had to decide whether the stimulus in the cued modality had been presented for a short or long period. Both responses required a key press on a response pad (Black Box ToolKit). The stimulus-response mapping was counterbalanced across participants. A trial was terminated when a response was given or after 2,500 ms. The response-cue interval was constant at 1,500 ms. The equal importance of speed and accuracy was emphasized to the participants.

### Procedure

**Figure 3:**
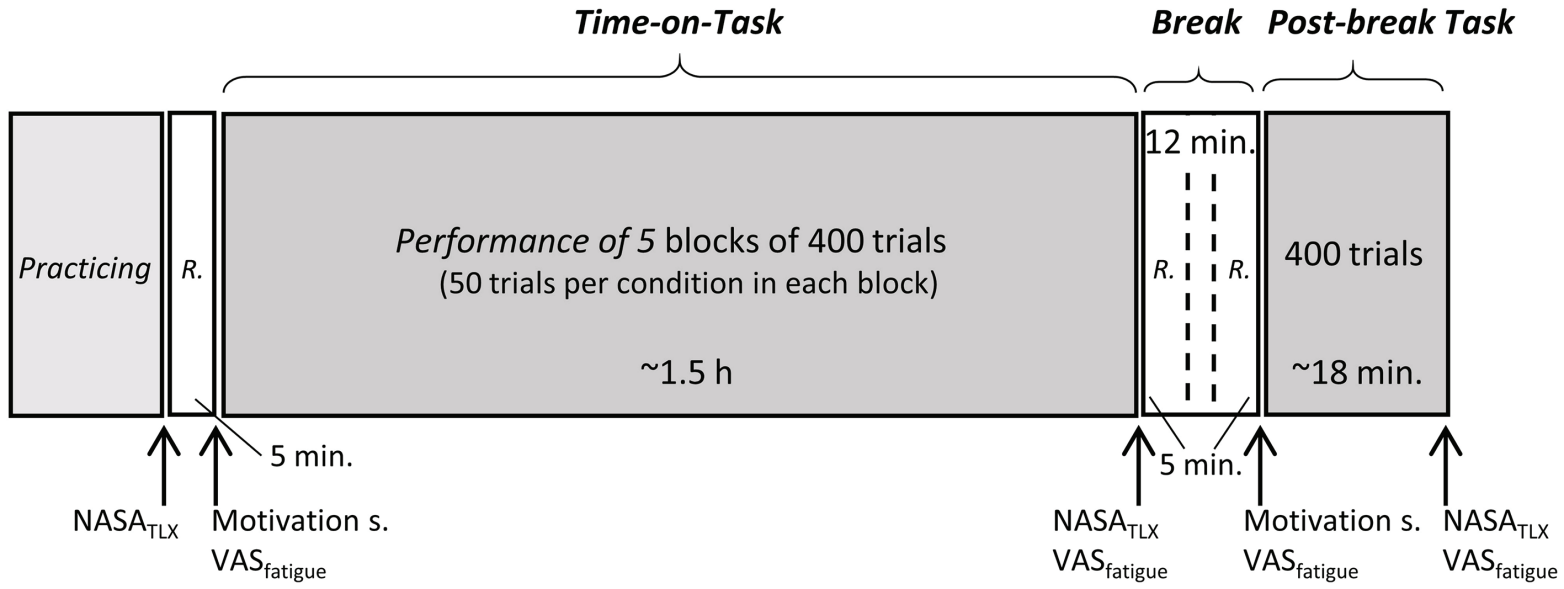
Schematic representation of the general procedure. NASA_TLX_, NASA Task Load Inventory; VAS_fatigue_, Visual Analogue Scale to assess subjective fatigue; Motivation s., motivation scale to assess subjective motivation to perform the task; R., Resting period with eyes opened and without large body movements.

Figure 3 schematizes the general procedure. The experimental sessions started at 10:00 am or 2:00 pm. To ensure appropriate wakefulness, participants with an evening-type chronotype were tested in the afternoon (see above for details of chronotype assessment). All participants were asked to get adequate sleep during the night prior to the experiment. Objective sleep duration was obtained using an actigraph with an integrated photopic light sensor (i.e., actiwatch; Philips Respironics: Actiwatch 2). The average sleep duration assessed by the actiwatch was 7.92 h (SD = 1.57). The mean self-reported sleep duration was 7.89 h (SD = 1.31). Participants were instructed to abstain from alcohol for 24 h before the experiment and from caffeine on the day of the experiment. Before the experiment, participants were briefed and provided written consent. Participants handed in their watches and mobile phones before starting the task.

#### Practice Trials and Time-on-Task

The experiment was conducted in a dark, sound-attenuated room. The first two blocks were 10 single-modality trials in each modality with visual feedback. This was followed by two additional blocks of 20 single-modality trials without feedback. The order of visual and auditory practice blocks was counterbalanced across participants. Finally, participants received two blocks of dual-modality practice trials: 32 trials with visual feedback and 32 trials without feedback. Immediately after the last practice block, participants completed the NASA Task Load Inventory (NASA_TLX_) assessing subjective task difficulties during the task. The NASA_TLX_ is a commonly used multidimensional, self-report measure that assesses perceived workload in terms of six components: mental demand, physical demand, temporal demand, overall performance level, effort, and frustration. The subscales are represented by bipolar scales ranging from 0 to 100 points divided into 21 gradations. After participants had completed the NASA_TLX_, the electrocardiographic (ECG) electrodes were set up (chest electrodes, Lead II) and then ECG was recorded during a 5-min pre-experiment resting period. During this period, participants were asked to sit comfortably with their eyes open and to avoid large body movements.

Additional questions about task-related motivation and subjective fatigue were administered before the start of the Time-on-Task manipulation. First, participants responded to the question “How motivated do you feel to do the task?” using a seven-point Likert-scale (1 = “Not motivated at all,” 7 = “Very highly motivated”). The scores for task-related motivation prior to the task was high (median = 6; mean = 5.52; range: 4–7) suggesting that participants were highly motivated to comply with the task instructions. Second, participants were asked to indicate their subjective fatigue on a printed visual analogue scale (VAS_fatigue_; 100 mm, “No fatigue at all” was printed on the left and “Very severe fatigue” on the right side of the scale).

After the pre-experiment resting period, the Time-on-Task period followed and the participants performed five blocks of 400 trials without a break (50 trials per condition in each experimental block). Stimuli were presented in pseudo-random order within each block. The exact duration of this Time-on-Task period depended on the participant’s reaction time. The mean duration was 1.55 h (SD = 0.1 h). When the Time-on-Task period ended, participants completed the NASA_TLX_ and the VAS_fatigue_ again.

#### Break Period

After the Time-on-Task period and filling in the questionnaires, participants were given a 12-min break. As previous studies with various task settings showed, a break duration around 10 min is long enough to restore cognitive resources and to improve the subsequent task performance (see e.g., Li et al., 2016; Lim et al., 2016; Lim and Kwok, 2016). Furthermore, to examine reactivity and recovery-related changes in HRV, we considered the comparison of 5-min-long intervals throughout the experiment (these recordings are advised to be identical in terms of duration.). For example during the break, the first 5 min were compared to the last 5 min of the ToT period (to examine recovery related changes in HRV), and the second 5 min period were compared with the first 5 min of the post-break block (to examine reactivity related changes after the break). Hence, the minimum duration of the break was 10 min in this concept. In the first and the last 5 min of the break period, ECG data were recorded. As in the pre-experiment rest period, participants were instructed to keep their eyes open and avoid large body movements. During the 2 min between ECGs participants simply had to wait in their chair. Participants reported their subjective fatigue using a VAS at the end of the break and their task-related motivation was measured using the same question as previously. The post-break task-related motivation scores were low (median = 3; mean = 3.43; range: 1–7), suggesting relatively low motivation to resume the task after the break. We did not use any motivational manipulation during the break and participants were unaware of how much longer they would be performing the task after the break.

#### Post-break Block of Trials

Following the break, participants performed an additional block of 400 trials (henceforth post-break block) to enable us to examine the impact of the break on performance. The task procedure remained exactly the same as before the break. After the experiment, in informal discussion, participants mentioned that they had expected a fairly long post-break experimental session. After completing the post-break block, participants reported their subjective workload and their fatigue level.

### Data Analysis

#### Analysis of the Subjective and Performance Measures

The data used in analyses are available at data.mendeley.com. Changes in subjective workload and fatigue were analyzed with paired-sample *t*-tests, and repeated measures ANOVAs (*r*ANOVA) with Administration Time (the administrations of the NASA_TLX_ scales) and Component (the six NASA_TLX_ scales) as within subject factors.

In order to examine the effect of Time-on-Task, the performance measures of the first five blocks of trials were subjected to repeated measures of ANOVA (*r*ANOVA) with Time-on-Task (ToT: 5 blocks of trials), Modality (visual versus auditory cue modality), Modality-transition (repetition versus switch trials), and Congruency (congruent versus incongruent) as within-subject factors. Three performance measures were entered at the *r*ANOVA: accuracy, reaction time of correct responses, and the linear integrated speed accuracy score (LISAS). The LISAS is a single, comprehensive index of performance combining speed, and accuracy measurements according to a formula proposed by Vandierendonck (2017): LISAS = RT + SD_RT_/SD_ER_ × ER, where RT is the participant’s mean reaction time, ER is the mean error rate, and SD_RT_ and SD_ER_ are the standard deviations of reaction time and error rate data, respectively, within a particular condition. The analysis of LISAS has a special relevance in fatigue research. When people become fatigued, they may switch performance strategies; some of them focus more on maintaining optimal RT at the expense of accuracy, the others might do the reverse. Therefore, LISAS probably is one of the best indicators of general effects of mental fatigue.

The Greenhouse-Geisser epsilon correction was applied when Mauchly’s test indicated violation of the sphericity assumption. The Bonferroni correction for multiple comparisons was applied. We analyzed break-related changes in performance using a *r*ANOVA with the same variables as above, but comparing just the last block (block 5) of the Time-on-Task period with the post-break block of trials.

#### Analysis of Heart Rate Variability

ECG data were recorded continuously throughout the experiment (not the practice blocks). Data were digitalized at a sampling rate of 1 kHz with 16 bit resolution using a CED 1401 Micro II analogue-digital converter device (CED, Cambridge, UK), and were stored on a computer in Spike2 (CED, Cambridge, UK) data format for off line analysis. Before analysis, the ECG signals were inspected visually and artifacts were removed. R-R intervals, in milliseconds, were extracted using the Spike2 software and analyzed further using Kubios HRV analysis software (version 3.1) to assess both the time and the frequency domain indices of HRV (Tarvainen et al., 2014). The low artifact correction option of the Kubios software was chosen and detected artifacts were replaced by estimates based on cubic spline interpolation. Spectral analysis was performed using the fast Fourier transformation routine provided by the software. The frequency and time indices that were analyzed were high frequency power (0.15–0.4 Hz; HF) and the root mean square of successive differences (RMSSD), respectively. High frequency power data were log-transformed because the distribution of the data deviated from normality.

## Results

### Subjective Fatigue and Workload

**Figure 4:**
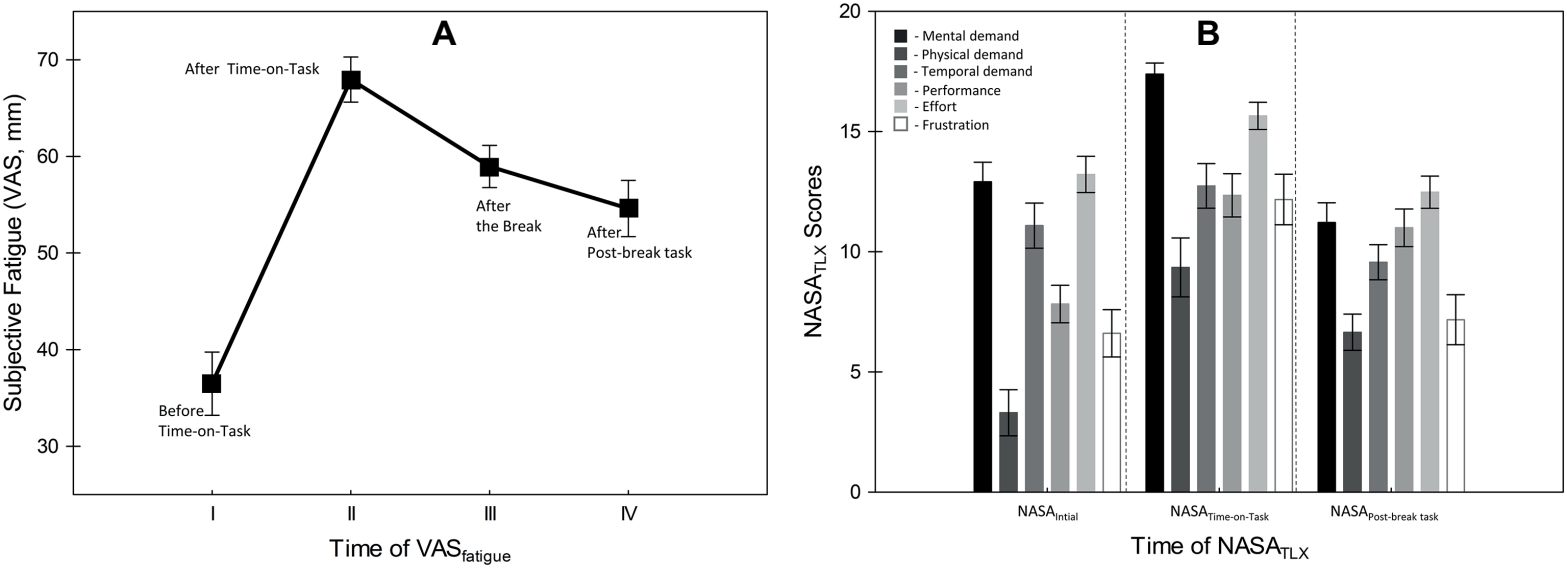
**(A)** Mean scores of the subjective fatigue (i.e., Visual Analogue Scale), and **(B)** subjective workload (i.e., NASA_TLX_) measurements obtained at different time points during the experiment. Error bars indicate within-subject SEM (O’Brien and Cousineau, 2015).

To investigate whether Time-on-Task (ToT) induced subjective fatigue, first, we analyzed the VAS_fatigue_ and NASA_TLX_ data. Figure 4 summarizes the results. The main effect of Administration time (pre ToT; after ToT; end-of-break, after-the-break) on the VAS_fatigue_ was highly significant (*F*(3, 66) = 23.82, *p* < 0.001, $\eta_{p}^{2}$ = 0.52). The corrected *post hoc* analyses showed that participants reported significantly higher fatigue after ToT than before (*t*(22) = −7.41, *p* < 0.001). After the break period, their fatigue level had significantly dropped, indicating some recovery (*t*(22) = 3.49, *p* < 0.001). Finally, the post-break block of trials no longer induced a significant increase in fatigue relative to the pre-break assessment (*t*(22) = 1.18, *p* = 0.52).

The analysis of subjective workload data (i.e., NASA_TLX_) also revealed a significant main effect of administration time (*F*(2, 44) = 21.09, *p* < 0.001, $\eta_{p}^{2}$ = 0.49), and component (*F*(5, 110) = 21.04, *p* < 0.001, $\eta_{p}^{2}$ = 0.49) and a significant interaction between the two factors (*F*(10, 220) = 4.97, *p* < 0.001, $\eta_{p}^{2}$ = 0.18). Participants rated the task as highly mentally demanding, on the basis of the practice trials (NASA_initial_ on Figure 4) and ratings of mental demand were even higher after ToT (*t*(22) = −4.95, *p* < 0.001; NASA_Time-on-Task_ on Figure 4), and finally significantly decreased in the post-break block (*t*(22) = 6.20, *p* < 0.001; NASA_Post-break task_ on Figure 4).

### Cognitive Performance in Time-on-Task

**Figure 5:**
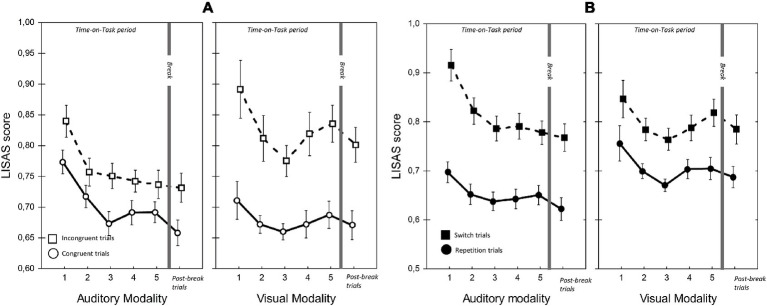
LISAS scores for visual and auditory modalities in the two Congruency **(A)** and Modality-transition conditions **(B)**. Error bars indicate within-subject SEM (O’Brien and Cousineau, 2015).

**Figure 6:**
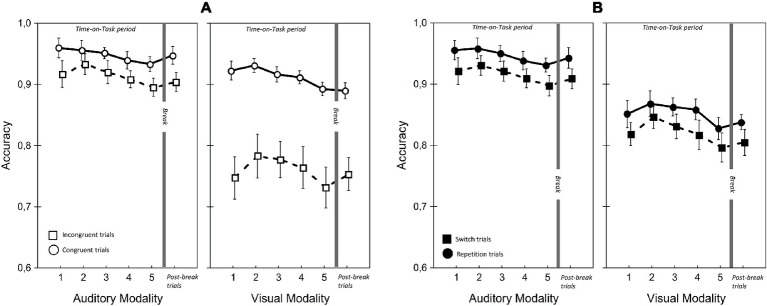
Accuracy rate for visual and auditory modalities in the two Congruency **(A)** and Modality-transition conditions **(B)**. Error bars indicate within-subject SEM (O’Brien and Cousineau, 2015).

**Figure 7:**
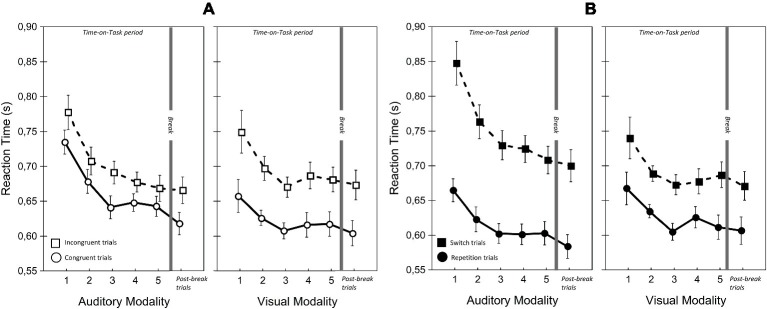
Reaction times for visual and auditory modalities in the two Congruency **(A)** and Modality-transition conditions **(B)**. Error bars indicate within-subject SEM (O’Brien and Cousineau, 2015).

Table 1, Figures 5–7 present the performance results. The main effects of modality-transition, and congruency were significant for all performance measures, replicating previous findings that switching attention between modalities has a cognitive cost (switching cost) and that incongruence in the durations of the visual and auditory stimuli induced response conflict (Lukas et al., 2014).

**Table 1:** Main effects and interactions yielded by a *r*ANOVA for the Time-on-Task (ToT) period.

Effects		LISAS scores		Accuracy		Reaction times	
	df	*F*	$\eta_{{}_{p}}^{{}^{2}}$	*F*	$\eta_{{}_{p}}^{{}^{2}}$	*F*	$\eta_{{}_{p}}^{{}^{2}}$
ToT (from block 1 to block 5)	4, 88	8.79***	0.28	6.05**	0.22	11.68***	0.35
Modality (visual or auditory)	1, 22	0.94	0.04	26.33***	0.54	4.54*	0.17
Modality-transition (switch or repetition)	1, 22	83.24***	0.79	26.39***	0.54	92.41***	0.81
Congruency (incongruent or congruent)	1, 22	60.54***	0.73	75.95***	0.77	56.30***	0.72
Modality × Modality-transition	1, 22	22.92***	0.51	0.01	0.00	36.39	0.62
Modality × Congruency	1, 22	12.92**	0.37	27.10***	0.55	8.22	0.27
Modality × Modality-transition × Congruency	1, 22	0.37	0.02	0.87	0.04	1.15	0.05
ToT × Modality	4, 88	3.18*	0.13	0.87	0.04	5.36**	0.20
ToT × Modality-transition	4, 88	4.63**	0.17	0.26	0.01	6.34**	0.22
ToT × Congruency	4, 88	2.32^m^	0.10	1.32	0.06	2.68^m^	0.11
ToT × Modality × Modality-transition	4, 88	5.36**	0.20	0.32	0.01	5.44**	0.20
ToT × Modality × Congruency	4, 88	3.86*	0.15	0.34	0.01	1.68	0.07
ToT × Modality-transition × Congruency	4, 88	1.04	0.04	2.10	0.09	0.12	0.00
ToT × Modality × Modality-transition × Congruency	4, 88	0.39	0.02	1.93	0.08	0.52	0.02

* p < 0.05;

** p < 0.01;

*** p < 0.001;

m *p = 0.06–0.08*.

There were significant main effects of Modality on accuracy and RT but they were in opposite directions. Accuracy was higher with auditory modality, but responses were faster with visual modality. This finding was due to the greater RT cost of switching for auditory modality than for visual modality. A similar phenomenon was reported by Lukas et al. (2014); we discuss this aspect of our data in more detail below. There was, however, no significant main effect of Modality on LISAS, showing that there was no overall performance advantage for one modality.

There was a significant main effect of ToT on all performance variables; performance appeared to improve during the first two blocks of trials and decline slightly in the last two blocks. Corrected pairwise comparisons confirmed improvements between blocks 1 and 2 in RT (*t*(22) = 3.52, *p* < 0.02) and LISAS (*t*(22) = 3.52, *p* < 0.05) but the decline in the last blocks reached significance only for accuracy (block 2 vs. block 5: *t*(22) = 5.02, *p* < 0.05).

Importantly, and in line with our prediction, there were significant ToT × Modality interactions for RT and LISAS. In the case of RT, the interaction reflected continuous improvement in auditory modality during the first three blocks, (block 1 vs. block 3: *t*(22) = 5.14, *p* < 0.05; block 3 vs. block 5: *t*(22) = 0.77, *p =* 1.0) combined with an improvement in visual modality until block 3 followed by a non-significant slowing down (block 1 vs. block 3: *t*(22) = 3.42, *p* < 0.05; block 3 vs. block 5: *t*(22) = −1.05, *p* = 0.31). Importantly, with LISAS the ToT × Modality interaction revealed that performance declined differently with increasing Time-on-Task for auditory and visual stimuli. LISAS showed no significant change from block 3 to block 5 in auditory trials (block 3 vs. block 5: *t* = −0.15, *p* = 0.88), but significantly decreased in visual trials (block 3 vs. block 5: *t* = − 3.34, *p* < 0.05). These findings imply that visual modality is generally more sensitive to the detrimental effects of fatigue (i.e., Time-on-Task) than the auditory modality when the task demands temporal operations.

We also found a significant three-way ToT × Modality × Congruency interaction for LISAS. Further analysis of this interaction (ANOVAs and pairwise comparisons) provided support for our second hypothesis, that in visual trials the congruency effect (i.e., the interfering effect of duration-incongruent irrelevant stimuli) increased with ToT. Separate analyses of the two modalities showed significant ToT × Congruency interactions for both modalities (visual: *F*(4, 88) = 2.93, *p* < 0.05, $\eta_{p}^{2}$ = 0.12; auditory: *F*(4, 88) = 3.18, *p* < 0.05, $\eta_{p}^{2}$ = 0.13), albeit for different reasons. In the case of visual trials with an incongruent auditory distractor, there was a significant main effect of ToT (*F*(4, 88) = 5.20, *p* < 0.01, $\eta_{p}^{2}$ = 0.19), reflecting an improvement between blocks 1 and 3 (*t*(22) = 3.66, *p* < 0.01), followed by a decline between blocks 3 and 5 (*t*(22) = −3.99, *p* < 0.01). In contrast, there was no significant change in LISAS over the five blocks in the case of visual trials with a congruent auditory distractor (*F*(4, 88) = 2.32, *p* = 0.12, $\eta_{p}^{2}$ = 0.09). The finding most relevant to our hypothesis is that, in the visual modality, the congruency effect increased in the second part of ToT, as participants became fatigued. As stated above, there was also a significant ToT × Congruency interaction for auditory trials, but in this case it reflected a larger improvement, over blocks 1–3, in performance on trials with a congruent visual distractor (block 1 vs. block 2: *t*(22) = 3.96, *p* < 0.01; block 2 vs. block 3: *t*(22) = 3.48, *p* < 0.01) than trials with an incongruent visual distractor (i.e., performance did not improve after block 2; block 2 vs. block 3: *t*(22) = 0.52, *p* = 1.0). There was, however, no significant change in LISAS in either congruent (*t*(22) = −1.15, *p =* 1.0) or incongruent auditory trials (*t*(22) = 0.9, *p* = 1.0) in the second part of the ToT. In other words, we found no evidence that the interfering effect of visual distractors on performance of auditory trials increased with Time-on-Task.

In contrast to the greater vulnerability of vision to cross-modal interference, we found that the modality switching costs (i.e., difference between switch and repetition trials) were similar or greater when switching to audition. In line with Lukas et al. (2014), the RT and LISAS switching costs were found to be larger when switching to auditory modality than when switching to visual modality. However, the large cost of switching to auditory modality did not increase with ToT. Although there was a ToT × Modality × Modality-transition interaction, it was not due to an increase in switching cost to auditory modality during the course of ToT. In fact, as separate analyses revealed, the interaction reflected two effects. First, in the case of auditory but not visual trials, RT and LISAS improved more during blocks 1–3 on switch trials than repetition trials (ToT × Modality-transition for the first three blocks; RT: *F*(2, 44) = 8.79, *p* < 0.01, $\eta_{p}^{2}$ = 0.29; LISAS: *F*(2, 44) = 10.52, *p* < 0.001, $\eta_{p}^{2}$ = 0.32). Second, between blocks 3 and 5 the decline in performance was greater for visual switch trials than for the auditory switch trials (ToT × Modality on switch trials from block 3 to block 5; Reaction times: *F*(2, 44) = 8.79, *p* < 0.01, $\eta_{p}^{2}$ = 0.29; LISAS score: *F*(2, 44) = 3.83, *p* < 0.05, $\eta_{p}^{2}$ = 0.15). In summary, we found that the cost was greater when participants switched to the auditory modality, but the cost of switching from visual to auditory modality nevertheless remained robust to the detrimental effects of Time-on-Task.

Finally, we also performed bivariate correlation analyses and ANCOVAs to explore the relationships between the ToT-related changes in cognitive performance and the changes in HRV and the subjective variables. No significant associations were found.

### Heart Rate and Heart Rate Variability in Time-on-Task

**Figure 8:**
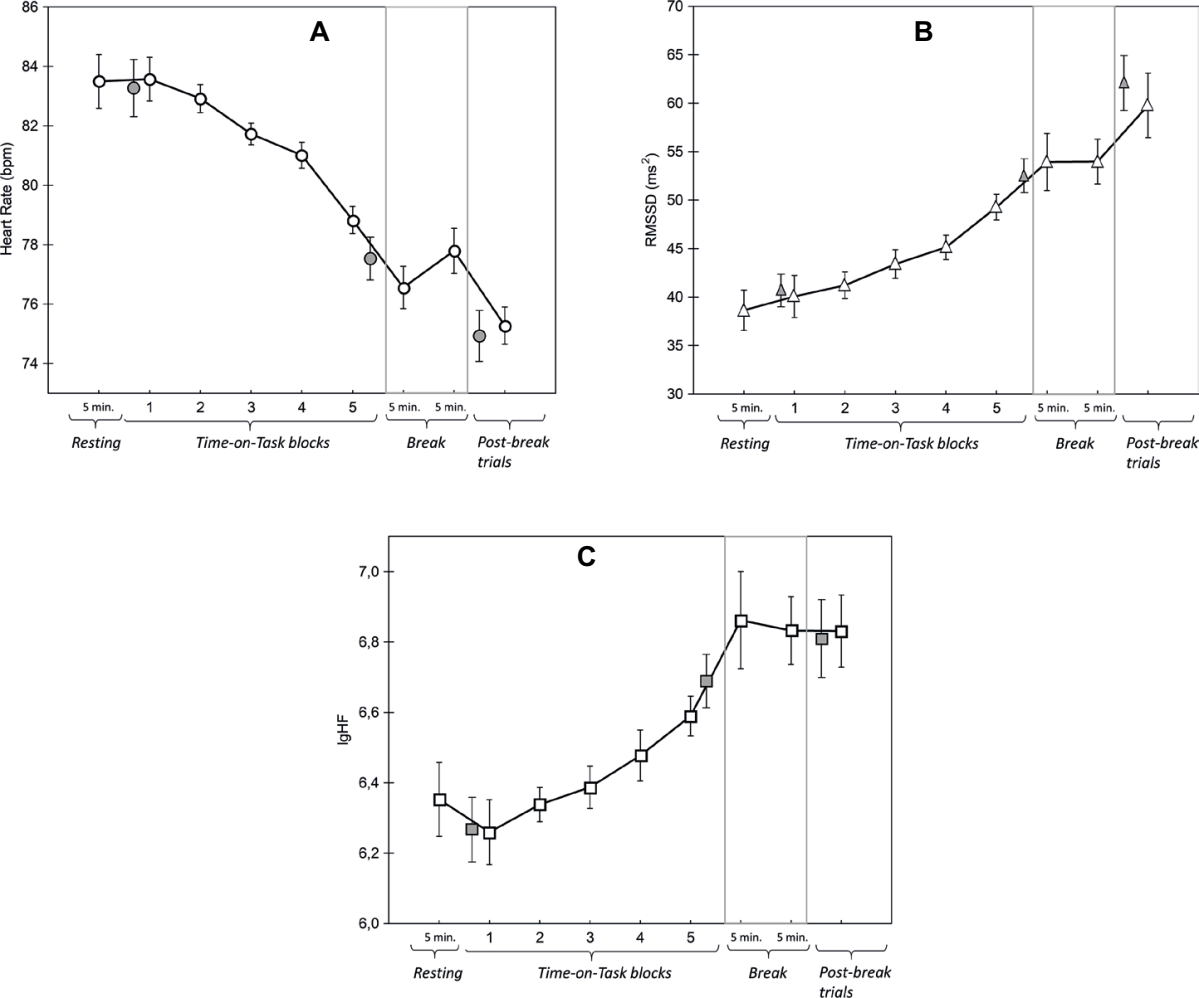
Results of mean Heart Rate **(A)** and two Heart Rate Variability measures **(B,C)** over the whole course of the experiment. The gray-filled symbols in each graph represent the data in the first 5 min of block 1, the last 5 min of block 5, and the first 5 min of the post-break block (from left to right). Please note that these 5-min-long periods were compared with the resting and break periods to calculate reactivity and recovery related changes in heart rate and heart rate variability (see the text for details). Error bars indicate within-subject SEM (O’Brien and Cousineau, 2015).

Changes in heart rate (HR) are shown in Figure 8. We found no difference between HR during the pre-experiment rest period and the first 5 min of the first block (*t*(22) = 0.38, *p* = 0.70). In contrast, there was a main effect of Time-on-Task (*F*(4, 88) = 13.66, *p* < 0.001, $\eta_{p}^{2}$ = 0.38, linear trend: *F*(4, 88) = 19.72, *p* < 0.001, $\eta_{p}^{2}$ = 0.47) with *post hoc* tests revealing that HR only started to significantly decrease in block 4 (block 1 vs. block 4: *t*(22) = 2.61 *p* = 0.16; block 4 vs. block 5: *t*(22) = 4.86, *p* < 0.01). HR did not significantly decline further in the break period (last 5 min of block 5 vs. the first 5 min of the break: *t*(22) = 1.28, *p* = 0.21). Finally, in the post-break block, HR was significantly lower than in the break period (last 5 min of the break vs. the first 5 min of post-break block: *t*(22) = 2.59, *p* < 0.05).

The two vagal-mediated HRV components (RMSSD, HF) followed similar trends to HR. First, HRV did not significantly increase when participants started the ToT period (pre-experiment rest vs. the first 5 min of block 1; RMSSD: *t*(22) = −0.67, *p* < 0.51; HF: *t*(22) = 0.79, *p* = 0.44). Second, there were significant main effects of ToT on RMSSD (*F*(4, 88) = 6.12, *p* < 0.01, $\eta_{p}^{2}$ = 0.22) and HF (*F*(4, 88) = 3.61, *p* < 0.05, $\eta_{p}^{2}$ = 0.14). Both HRV components increased significantly and linearly during ToT. The *post hoc* tests showed that RMSSD was significantly higher in block 5 than the previous four blocks (e.g., block 1 vs. block 4: *t*(22) = −1.83, *p* = 0.81, block 5 vs. block 1: *t*(22) = −3.05, *p* = 0.06; block 5 vs. block 2: *t*(22) = −3.76, *p* < 0.05; block 5 vs. block 4: *t*(22) = −3.39, *p* < 0.05). The changes in HF during ToT were smaller; corrected pairwise comparisons yielded only one marginally significant difference between blocks (block 5 vs. block 2: *t*(22) = −2.80, *p* = 0.07).

### Break-Related Changes in Cognitive Performance and Heart Rate Variability

The comparison of the post-break block with block 5 implied that the break did not result in a significant overall improvement in performance. More specifically, participants’ overall cognitive performance was neither significantly more accurate nor faster after the break than in the last block of the ToT period. There was, however, a significant post-break improvement in RT, but only on trials where stimulus duration was congruent between modalities. The analysis of RTs showed that responses on congruent trials were significantly faster after the break than before (Block × Congruency: *F*(1, 22) = 6.46, *p* < 0.05, $\eta_{p}^{2}$ = 0.23). The post-break improvement in LISAS was greater in congruent auditory trials than in the other conditions (Block × Modality × Congruency: *F*(4, 88) = 7.29, *p* < 0.05, $\eta_{p}^{2}$ = 0.25; Block × Congruency for auditory trials: *F*(1, 22) = 8.46, *p* < 0.01, $\eta_{p}^{2}$ = 0.28; block 5 vs. post-break block for congruent auditory trials: *t*(22) = 2.40, *p* < 0.05). Importantly, the modality difference in the congruency effect was unchanged after the break. In the post-break block, as in the ToT period, congruency effect was significantly greater for visual modality than for auditory modality (Modality × Congruency; Accuracy: *F*(1, 22) = 19.44, *p* < 0.001, $\eta_{p}^{2}$ = 0.47; LISAS: *F*(1, 22) = 6.06, *p* < 0.05, $\eta_{p}^{2}$ = 0.22; see Figure 5).

There were no significant changes in RMSSD and HF between the last block of ToT (block 5) and the first 5 min of the break period (recovery period; RMSSD: *t*(22) = −0.57, *p* = 0.57; HF: *t*(22) = −1.17, *p* = 0.25). In addition, neither HRV component changed significantly between the last 5 min of the break and the first 5 min of the post-break block (Reactivity after the break; RMSSD: *t*(22) = 1.93, *p* = 0.07; HF: *t*(22) = 0.10, *p* = 0.92).

The break-related improvement in performance was associated with the change in fatigue during the break (VAS_break-fatigue_). ANCOVAs with VAS_break-fatigue_ as a covariate yielded a significant Block × VAS_break-fatigue_ interaction for accuracy (*F*(1, 21) = 5.89, *p* < 0.05, $\eta_{p}^{2}$ = 0.22): Participants whose fatigue decreased most during the break performed more accurately in the post-break block than in block 5 of the ToT period. Importantly, the break-related changes in HRV were not significantly associated with durational incongruence between the two modalities.

## Discussion

Prolonged performance of a monotonous, attention-demanding task often induces performance deficits and a subjective feeling that can be labeled mental or cognitive fatigue. The aim of this study was to assess the potentially detrimental effects of mental fatigue on a task in which stimuli were presented in two modalities (visual and auditory). Although a variety of cognitive functions such as response selection, task-switching or sustained attention have been addressed in fatigue literature and have been tested under various conditions requiring more complex operations, most of the studies have used paradigms in which stimuli are only presented in a single modality (see e.g., Lorist et al., 2000; Boksem et al., 2005; Mizuno et al., 2011, 2014; Hopstaken et al., 2016; Puma et al., 2018). There has been very little use of bimodal stimuli in this research area, hence the main aim of this study was to address this gap by exploring potential fatigue-related changes in conflict between the visual and auditory modalities. There are two possible sources of conflict: the different responses facilitated by the visual and auditory stimuli and the sustained cognitive demand of switching attention between the two modalities. Numerous studies have shown that fatigue has a detrimental effect on response selection (e.g., Lorist et al., 2000; Van der Linden et al., 2003; Csathó et al., 2012; Möckel et al., 2015) and it can also compromise the efficiency of attentional-shifting mechanisms. In this study, we investigated these fatigue-sensitive cognitive operations in a bimodal temporal discrimination paradigm adapted from Lukas et al. (2014). Our predictions were derived from the modality appropriateness hypothesis, which posits that audition is given priority over vision during performance of tasks demanding temporal operations. Accordingly, based on the notion that fatigue can induce strategic adjustments in performance (Hockey, 1997), we expected that the visual modality would be more sensitive to the detrimental effect of Time-on-Task. In line with this expectation we found that both visual and auditory temporal discrimination performance initially improved with increasing Time-on-Task. After the third block of trials (i.e., after participants had been performing the task for about 45 min), however, performance in the two stimulus modality conditions started to differentiate. Performance on trials where the auditory stimulus was relevant remained unchanged during the rest of the ToT period, whereas performance on trials where the visual stimulus was relevant started to decrease. This pattern of findings suggests that as participants became tired, and probably started to disengage from the task, they attended more to the modality that better suited the temporal character of the task. This interpretation of the findings is in line with Hockey’s model of compensatory control model of fatigue (Hockey, 1997, 2011), which assumes that, in order to protect task activities from the detrimental effects of prolonged performance, impairments in performance are detected by an effort-monitoring system that forms part of the central executive and also facilitates the choice between two alternative strategies: exerting compensatory effort in order to maintain task performance or allowing performance to deteriorate in order to minimize effort. Effort can be minimized by neglecting secondary task activities and focusing on those that require less effort but still serve the main aims of the task performance. The performance impairment (in the second part of the Time-on-Task period and the pattern of cardiac activity (see the discussion below) suggest that participants chose the latter compensatory strategy. In other words, instead of investing more effort in the task, which would have manifested as a reduction in HRV, they disengaged and attended less to visual stimuli, because temporal discriminations are harder in the visual modality than the auditory modality.

The findings on modality dominance in relation to the two different types of trial manipulations (congruency and trial-transition) are in line with those of Lukas et al. (2014). Specifically, we found reversed modality effects for the two different trial manipulations. This implies that there was less difference between incongruent and congruent trials when the auditory stimulus was relevant than when the visual stimulus was relevant. In other words, visual information interfered less with processing of an auditory target than vice versa. This finding replicates the results of Lukas et al. (2014) and is in line with the modality appropriateness hypothesis of auditory dominance in temporal tasks. In addition, and importantly in the context of this study, we found that the congruency effect increased with Time-on-Task in the case of visual trials but not auditory trials. Again, this is in accordance with the prediction that response conflict between visual and auditory stimuli tends to be enhanced when the efficiency of the fatigue sensitive control processes drops as with the increasing time spent with the task (e.g., Csathó et al., 2012).

Like Lukas et al. (2014), we found that whereas changes in the congruency effect suggested that the visual modality was more sensitive to fatigue in the context of a temporal discrimination task, the costs of switching to the auditory modality (in RT and LISAS) were greater than the costs of switching to the visual modality; in other words the visual-to-auditory transition was more difficult relative to visual repetition trials than the auditory-to-visual transition relative to the auditory repetition trials.

The asymmetric switch cost seems not to be merely induced by the modality differences in the time required for reconfiguration (i.e., preparation time for the next trial), because, in their second experiment, Lukas et al. (2014) observed that an increasing cuing interval did not alter the asymmetric switch cost between the modalities. A more plausible explanation is that trials with visual target required a stronger attentional selection, making it more difficult for participants to subsequently switch their attention from vision to audition (Karle et al., 2010). In line with this interpretation, performance on the visual repetition trials was indeed worse than on the auditory repetition trials (see Figure 5 for LISASs) indicating that visual trials generally placed greater demands on cognitive operations and required a higher level of selective attention. In addition, asymmetric switch cost is frequently interpreted by proactive interference in working memory caused by the switching between trials with different task features (e.g., between the visual and auditory trials in the current study; Allport et al., 1994, Wylie and Allport, 2000; Vandierendonck et al., 2010). This theory generally proposes that switch costs are attributable to conflicts arising from working memory due to the performance of the preceding trial with different task feature. Allport et al. (1994) suggest that the proactive interference from trials with non-dominant features (or task sets) is greater than that from the dominant features because the non-dominant features require stronger working memory activation. Disengaging from the trials with non-dominant stimulus features would therefore be more difficult, and the switch cost to trials with dominant features would be large (Hunt and Klein, 2002). Applying the interference theory to the current results means that we can assume that the higher switch cost for dominant, auditory modality may be caused by the higher level of cognitive control processes required to deal with the persisting activation of the preceding visual modality trial in working memory (Badre and Wagner, 2006). Importantly, the cost of switching to auditory modality and performance on auditory switch trials remained unchanged throughout ToT, whereas performance on visual switch trials was worse in the second part of ToT. This finding provides further support for the conclusion that participants’ generally prioritized auditory trials over visual trials, so that despite the high cost of switching to audition, even performance on auditory switch trials was insensitive to the detrimental effects of fatigue.

Cognitive performance declined during Time-on-Task as fatigue increased and did not recover after the break. In other words, fatigue had a detrimental effect on cognitive performance that was not reversed by a short rest period. Participants’ motivation to continue the task was also low after the break. Although we found a break-related improvement for the auditory condition, this only occurred in the congruent trials. That the break only improved performance if a relevant auditory stimulus and irrelevant visual stimulus facilitated the same response (i.e., when they were congruent) suggests that participants were more vigilant after the break, but this enhanced vigilance was not accompanied by more efficient attention selection (i.e., a reduction in the congruency effect), and attention shift abilities (i.e., a reduction in switch cost).

Regarding the physiological measures, we interpreted HRV as an index of vagus-mediated cardiac mechanisms underlying task performance. It is recognized that changes in vagal activity indirectly reflect the activity of the LC-NE system (Mather et al., 2017), which has also been suggested to play a role in the effects of mental fatigue (Hopstaken et al., 2015a,b).

We observed that both HRV components increased as a function of Time-on-Task, which is in accordance with our prediction that parasympathetic inhibition *via* the vagus nerve increases as Time-on-Task increases and indirectly suggests that prolonged task performance was associated with a reduction in LC-NE activity.

The linear increase in vagus-mediated HRV functions seems to support our conclusion that during the second part of the experiment participants did not exert extra effort to compensate for fatigue induced performance deficit. In other words, the increasing vagal inhibition as a function of Time-on-Task, and the continuous decrease in HR do not suggest compensation *via* an increase in cardiac activity (i.e., *via* vagal withdrawal). Physiological compensatory mechanisms (e.g., increased sympathetic activation) are an important component of Hockey’s fatigue model with respect to the strategy of increasing the effort budget to protect performance. Our HR and HRV data seem to support another strategy postulated by Hockey, namely the task disengagement strategy whereby the goal of task is partly maintained but at a lower performance level.

In the present study, subjective levels of fatigue, performance, and the physiological indicators did not show direct significant associations with each other. In the fatigue literature, this is a well-known phenomenon that has been attributed to between-subject baseline differences in response styles, performance, and basic physiological parameters (e.g., Nunan et al., 2010).

Part of the lack of direct associations may also be due to differential trends of the various measures. Specifically, performance seemed to show a quadratic trend in which performance initially increased, probably due to learning, but at some point started to decrease because of fatigue. HRV, on the other hand, showed a linear pattern, with increasing values as Time-on-Task increased. A similar linear pattern was found for task disengagement in earlier studies (see e.g., Hopstaken et al., 2015a,b). Such pattern of differential trends also suggests that the participants’ ability for temporal discrimination of the stimuli was rather robust to changes in vagal activity. Initially, it seems that one can maintain and even improve performance, despite indications of decreasing engagement, as shown by the increasing HRV. Yet, when a maximum level of performance or learning has been reached, further disengagement (higher HRV) is accompanied with decreasing performance in the more difficult trial conditions (e.g., visual trials with an incongruent auditory distractor).

The robustness of temporal discrimination to ToT-related changes in vagal activity is in line with the findings of recent studies showing that vagus-mediated HRV is associated with temporal perception mainly if the temporal stimulus per se is a source of arousal (e.g., an emotional picture; Piovesan et al., 2018; Ogden et al., 2019). Accordingly, in the current study, we used emotionally neutral stimuli without a stimulatory effect on arousal and probably having a weaker association with vagal-mediated HRV functions.

Although the main scope of this study was to investigate cross-modal temporal interference under mental fatigue, our findings might also be relevant for the ongoing debate about the cognitive architecture of timing mechanisms being centralized and modality independent versus distributed and modality-specific (Levitan et al., 2015; Motala et al., 2018). The additional cost we found that was associated with switching from vision to audition relative to the auditory repetition trials (for comparison, the difference between audition-vision switches and visual repetition trials was much smaller) seem to be more in line with the notion that there are distributed, modality-specific timing mechanisms. On the other hand, the differential switch costs do not necessarily have to reflect different timing mechanisms. For example, in line with the modality appropriateness hypothesis, the specific findings on switch costs may also be due to differential processing load of the modalities. Visual stimuli might simply provide less reliable time-relevant information for an efficient temporal discrimination than auditory stimuli, and thus, would require more processing resources (e.g., time) and/or higher cognitive effort in order to retrieve the information that has to be feed into the timing mechanism (irrespectively whether such a mechanism is centralized or distributed). This initial modality difference in temporal information preciosity might strongly affect performance in trials with modality transition, for example, *via* the proactive interference effects as discussed above extensively. In addition, recent studies (Murai and Yotsumoto, 2016, 2018) suggest that the role of modalities in temporal processing is not necessarily manifested in a conflict between the modalities. In contrast, the brain might incorporate the multisensory information (i.e., visual and auditory information) to improve timing sensitivity. The current study focused on the fatigue sensitivity of the conflict between auditory and visual modalities but future studies might investigate how fatigue affects the multisensory integration of temporal information.

To conclude, we found additional support for the phenomenon, first reported by Lukas et al. (2014), that individuals’ perception is dominated by auditory rather than visual stimuli when a temporal discrimination task demands attentional control processes. The key finding of our study is that in the visual modality, but not the auditory modality we found that the congruency effect increased as a function of Time-on-Task. The cost of switching attention was higher when switching to the auditory modality, but remained unchanged during the course of the experiment, providing further evidence for the relative insensitivity of the auditory modality to the detrimental effects of fatigue due to prolonged task performance. The fatigue-related changes in cognitive performance were not reversed by a short break, suggesting that fatigue-related performances are not transient and cannot be reversed by a brief rest period. The vagus-mediated HRV results indirectly suggest that participants’ LC-NE activity decreased during prolonged task performance, although this physiological index of fatigue did not covary with performance.

## Data Availability Statement

Data repository: https://data.mendeley.com/datasets/84687fys3r/draft?a=27d04c2c-a892-4a93-94f9-5e9f7e11338d.

## Ethics Statement

The studies involving human participants were reviewed and approved by the Ethics Committee of the Medical School, the University of Pécs (nr. 7698). The patients/participants provided their written informed consent to participate in this study.

## Author Contributions

AM and ÁC designed and performed the experiments, analyzed data and wrote the paper. DL interpreted the data and wrote the paper. KT performed the experiments and analyzed data. All authors discussed the results and implications and commented on the manuscript at all stages.

### Conflict of Interest

The authors declare that the research was conducted in the absence of any commercial or financial relationships that could be construed as a potential conflict of interest.
